# Free radical scavenging decreases endothelin‐1 excretion and glomerular albumin permeability during type 1 diabetes

**DOI:** 10.14814/phy2.13055

**Published:** 2016-12-30

**Authors:** Mohamed A. Saleh, Carmen De Miguel, David I. Stevens, Pamela K. Carmines, David M. Pollock, Jennifer S. Pollock

**Affiliations:** ^1^ Medical College of GeorgiaAugusta UniversityAugustaGeorgia; ^2^ Division of Clinical PharmacologyDepartment of MedicineVanderbilt University Medical CenterNashvilleTennessee; ^3^ Department of Pharmacology and ToxicologyFaculty of PharmacyMansoura UniversityMansouraEgypt; ^4^ Cardio‐Renal Physiology & MedicineDivision of NephrologyDepartment of MedicineUniversity of Alabama at BirminghamBirminghamAlabama; ^5^ Department of Cellular and Integrative PhysiologyUniversity of Nebraska Medical CenterOmahaNebraska

**Keywords:** Diabetes, ET‐1, glomerular permeability, tempol

## Abstract

Increased renal endothelin‐1 (ET‐1) production and an ET_A_ receptor‐dependent increase in glomerular albumin permeability (P_alb_) accompany type 1 diabetes mellitus (T1D). We hypothesized that T1D‐induced oxidative stress contributes to renal ET‐1 production and glomerular P_alb_. Male rats with streptozotocin‐induced T1D were provided free access to drinking water without additives (T1D rats) or containing the free radical scavenger tempol (1 mmol/L; T1D+Tempol). After 3 weeks, T1D+Tempol rats displayed lower urinary excretion of thiobarbituric acid reactive substances and glomerular superoxide production (dihydroethidium staining) compared to T1D rats. Urinary ET‐1 excretion and inner medullary (but not cortical or outer medullary) prepro‐ET‐1 mRNA expression were lower in the T1D+Tempol group than in the T1D group. P_alb_, measured as the change in volume of isolated glomeruli upon exposure to oncotic gradients of albumin, was significantly lower in the T1D+Tempol group than in the T1D group. Tempol treatment did not alter protein excretion or creatinine clearance. These data support the postulate that oxidative stress contributes to glomerular P_alb_ and renal ET‐1 production during the early phase of type 1 diabetes.

## Introduction

Despite widespread use of renoprotective therapies over the past 30 years, patients with type 1 diabetes (T1D) and persistent macroalbuminuria remain at high risk for developing end‐stage renal disease (Rosolowsky et al. 2011). The need for more effective therapies continues to fuel active investigation, with activation of the endothelin (ET) system among the many events suspected to contribute to the renal complications of T1D (Vallon and Komers 2011). Elevated extracellular glucose levels stimulate ET‐1 release from endothelial cells (Yamauchi et al. 1990; Khamaisi et al. 2008), and rodent models of T1D display increased renal ET‐1 expression at both the mRNA (Hargrove et al. 2000; Chiu et al. 2008) and protein levels (Chen et al. 2005), as well as elevated urinary ET‐1 excretion (a marker of intrarenal ET‐1 generation) (Hocher et al. 1998; Sasser et al. 2007). ET_A_ receptor antagonism prevents albuminuria for at least 10 weeks in rats with streptozotocin (STZ)‐induced T1D (Sasser et al. 2007), and recent clinical trials suggest that blockade of the ET system may reduce the progression of diabetic nephropathy as evidenced by diminished proteinuria (Kohan et al. 2015). Therefore, a more in‐depth understanding of the mechanisms through which the renal ET system is activated early during the course of the disease could better inform the therapeutic use of ET antagonists in patients with T1D, potentially including approaches that minimize or prevent development of diabetic nephropathy.

Chronic hyperglycemia in diabetes is widely recognized to provoke systemic and renal oxidative stress (Singh et al. 2011). For example, our previous studies demonstrated that rats with STZ‐induced T1D display marked increases in oxidative stress markers (plasma TBARS and urinary excretion of both TBARS and H_2_O_2_), as well as increased NADPH oxidase subunit expression and activity that contributes to accelerated renal production of superoxide anion (O_2_^•–^) (Ishii et al. 2001; Sasser et al. 2007; Yang et al. 2009, 2010; Troncoso Brindeiro et al. 2012). Such pro‐oxidant processes and/or a defect in antioxidant defense mechanisms could contribute to the pathogenesis of diabetic nephropathy (Stanton 2011). Indeed, antioxidants suppress high glucose‐induced extracellular matrix protein synthesis by cultured rat mesangial cells (Zhang et al. 2012) and prevent glomerular and renal hypertrophy (Kashihara et al. 2010), albuminuria (Peixoto et al. 2009), and glomerular transforming growth factor (TGF)‐*β*1 expression (Jiao et al. 2011) in rodent models of T1D. Although large clinical trials have yielded mixed results (Stanton 2011; Di Marco et al. 2015), antioxidants have been reported to normalize glomerular hyperfiltration (Hernandez‐Marco et al. 2009) and albuminuria (Giannini et al. 2007) in young patients with T1D. O_2_^•–^ accumulation contributes to several forms of glomerular injury (Datta et al. 2006; Leibowitz et al. 2016), including that accompanying T1D (Gorin et al. 2015), and has been implicated in the glomerular albumin permeability (P_alb_) defects induced by tumor necrosis factor alpha (TNF‐*α*) (Mccarthy et al. 1998), TGF‐*β* (Sharma et al. 2000), xanthine oxidase (Dileepan et al. 1993) and asymmetric dimethyl‐l‐arginine (ADMA) (Sharma et al. 2009). These actions illustrate the possible role of O_2_^•–^ in directly or indirectly impacting the glomerular filtration barrier during T1D.

The renal endothelin system and oxidative stress likely interact in contributing to the glomerular changes associated with T1D. Chen et al. (2000) reported that endogenously derived reactive oxygen species (ROS) enhance ET‐1 production by glomeruli from rats with T1D and that chronic treatment with ROS scavengers markedly suppresses glomerular ET‐1. The same group subsequently reported that exogenous ET‐1 stimulates ROS production, specifically O_2_^•–^, by glomeruli isolated from rats with T1D (Lee et al. 2010). Hence, it is not clear whether ROS promote glomerular ET‐1 production or vice versa (or both) during the early stage T1D, potentially initiating a vicious cycle of ROS and ET‐1 production.

We previously reported that chronic ET‐1 infusion in normal rats increases glomerular P_alb_ (Saleh et al. 2010) through an ET_A_‐dependent mechanism. Moreover, rats with STZ‐induced T1D display not only an increase in renal ET‐1 production (Hocher et al. 1998; Chen et al. 2005; Sasser et al. 2007; Chiu et al. 2008) but also a substantial increase in glomerular P_alb_ (Saleh et al. 2011; Fan et al. 2015)that can be prevented or reversed by treatment with an ET_A_ receptor antagonist (Saleh et al. 2011). Although these observations indicate that activation of the ET system increases glomerular P_alb_ in T1D, the interaction between these events and the oxidative stress accompanying T1D remains unexplored. We previously reported that ET_A_ receptor antagonism blunted T1D‐induced albuminuria and renal inflammation, but did not impact indices of oxidative stress (Sasser et al. 2007). Therefore, we hypothesized that oxidative stress contributes to renal ET‐1 synthesis and glomerular P_Alb_ during the early stage of T1D. The validity of this postulate was explored by determining the impact of the free radical scavenger tempol on renal ET‐1 synthesis and urinary ET‐1 excretion, as well as glomerular P_alb_ and urinary albumin excretion, in rats with STZ‐induced T1D. Animals were studied 3 weeks after inducing T1D, as the increases in both glomerular P_alb_ and urinary protein excretion evident at this time point can be fully prevented by chronic treatment with an ET_A_ receptor antagonist (Saleh et al. 2011).

## Methods

### STZ‐induced model of T1D in the rat

All protocols were approved by the Institutional Animal Care and Use Committee of the Medical College of Georgia at Augusta University, in accordance with NIH Guidelines for the Care and Use of Laboratory Animals. T1D was induced in male Sprague–Dawley rats (250–275 g), purchased from Harlan Laboratories (Indianapolis, IN), by intravenous injection of 65 mg/kg STZ (Sigma‐Aldrich, St. Louis, MO) dissolved in sterile saline as previously described (Sasser et al. 2007). The resulting hyperglycemia was moderated by subcutaneous implantation of an insulin pellet (Linplant^®^, LinShin Canada, Inc., Scarborough, Canada) that released insulin at a rate of ~1 unit per day. Rats were provided free access to food and to drinking water that contained either no additives (T1D rats, *n *=* *12) or 1 mmol/L tempol (Sigma‐Aldrich; T1D+Tempol rats, *n *=* *12). This tempol treatment regimen has been shown to be effective in decreasing renal oxidative stress in rats with STZ‐induced T1D for periods of 2–6 weeks (Asaba et al. 2007; Rodriguez et al. 2011; Luan et al. 2012; Troncoso Brindeiro et al. 2012). Three weeks after onset of T1D, each rat was placed in a metabolic cage for 2 days, with urine samples collected on the second day. Rats were then anesthetized (sodium pentobarbital, 65 mg/kg, i.p.), arterial blood was collected, and kidneys were removed for glomerular isolation or dissection into cortex, outer medulla, and inner medulla.

### Isolation of glomeruli

Glomeruli were isolated by a graded sieving technique previously described (Misra 1972). The resulting decapsulated glomeruli were devoid of afferent and efferent arterioles and contained less than 5% tubular contamination. The glomeruli were suspended in PBS, and utilized for dihydroethidium (DHE) staining and P_alb_ measurements.

### Dihydroethidium staining

Aliquots (150 *μ*L) of glomerular suspension were transferred onto engraved glass slides and incubated 30 min in DHE (2 *μ*mol/L in PBS) at 37°C. Images were obtained by fluorescence microscopy (excitation wavelength = 488 nm; emission wavelength = 574–595 nm).

### Albumin permeability assay

The rationale and methodology for the determination of P_alb_ has been described in detail previously (Savin et al. 1992). Briefly, freshly isolated glomeruli (10–15 glomeruli/rat) were digitally photographed while suspended in 5% BSA phosphate buffer (pH 7.4), and again after changing the bath to 1% BSA. Glomerular volume changes induced by the oncotic pressure gradient change were mathematically converted to P_alb_, which can range from a value of zero (the filtration barrier is impermeable to albumin) to 1.0 (albumin moves across the glomerular filtration barrier at the same rate as water).

### Quantitative real‐time PCR (qRT‐PCR)

Renal cortical, outer medullary and inner medullary tissues were stored at −80°C until mRNA extraction using the Qiagen RNeasy RNA isolation kit and QIAshredder homogenizer columns (Qiagen, Valencia, CA). RNA concentration and purity were determined using a NanoDrop ND‐1000 Spectrophotometer (Thermo Scientific, West Palm Beach, FL). RNA (1 *μ*g) was reverse transcribed using the QuantiTect RT kit (Qiagen). A dilution of the resulting cDNA was used to quantify the relative content of mRNA by real‐time PCR (StepOnePlus^™^ Real‐Time PCR System, Applied Biosystems, Foster City, CA) using commercially available QuantiTect primer assays to detect rat GAPDH and prepro‐ET‐1 (Qiagen; catalog numbers QT00371308, and QT00182546, respectively), with SYBR green as fluorescent probe. Fluorescence data were acquired at the end of extension. Expression of each target gene was calculated using the 2^−(ddC^_T_^)^ method and normalized to GAPDH expression.

### Plasma and urinary analyses

Creatinine concentration was measured in plasma and urine by the picric acid method adapted for microtiter plates (Allcock et al. 1998). Urinary ET‐1 concentration was measured by chemiluminescent immunoassay (QuantiGlo, R&D Systems, Minneapolis, MN). Thiobarbituric acid reactive substances (TBARS) in plasma and urine were quantified using an OXItek assay kit (ZeptoMetrix, Buffalo, NY). Urinary protein concentration was determined using the Bradford colorimetric method (Bio‐Rad Laboratories, Hercules, CA).

### Statistical analyses

All data are presented as mean ± SEM. Data obtained from T1D and T1D+Tempol groups were compared using unpaired Student's *t*‐test performed using GraphPad Prism Version 5.0 (GraphPad Software, La Jolla, CA). *P‐*values* *< 0.05 were considered to indicate statistical significance.

## Results

### Animal characteristics

Table 1 summarizes the metabolic cage data obtained from both groups of rats. Although body weight at the onset of diabetes was similar in both groups (T1D, 227 ± 5 g; T1D+Tempol, 230 ± 5 g), tempol treatment for the 3‐week duration of the study resulted in a lower final body weight than evident in nontreated T1D rats. Correspondingly, food intake in T1D+Tempol rats was less than that of T1D rats, as was water intake and urine flow. Neither creatinine clearance nor blood glucose concentration differed between groups.

**Table 1:** Characteristics of T1D and T1D+Tempol rats, 3 weeks after induction of diabetes

	T1D (*n* = 12)	T1D+Tempol (*n* = 12)
Body weight (g)	292 ± 3	268 ± 5**
Blood glucose concentration (mg/dL)	532 ± 23	524 ± 22
Food intake (g/day)	38 ± 2	30 ± 2*
Water intake (mL/day)	181 ± 19	116 ± 13*
Urine flow (mL/day)	174 ± 15	106 ± 11**
Creatinine clearance (mL/min)	1.22 ± 0.09	1.20 ± 0.08

Excretory data were derived from 24‐h urine collections in metabolic cages within 48 h of animal killing. Plasma samples were obtained under anesthesia immediately prior to killing.

*P < 0.05 and **P < 0.01 versus T1D.

### Oxidative stress parameters

**Figure 1:**
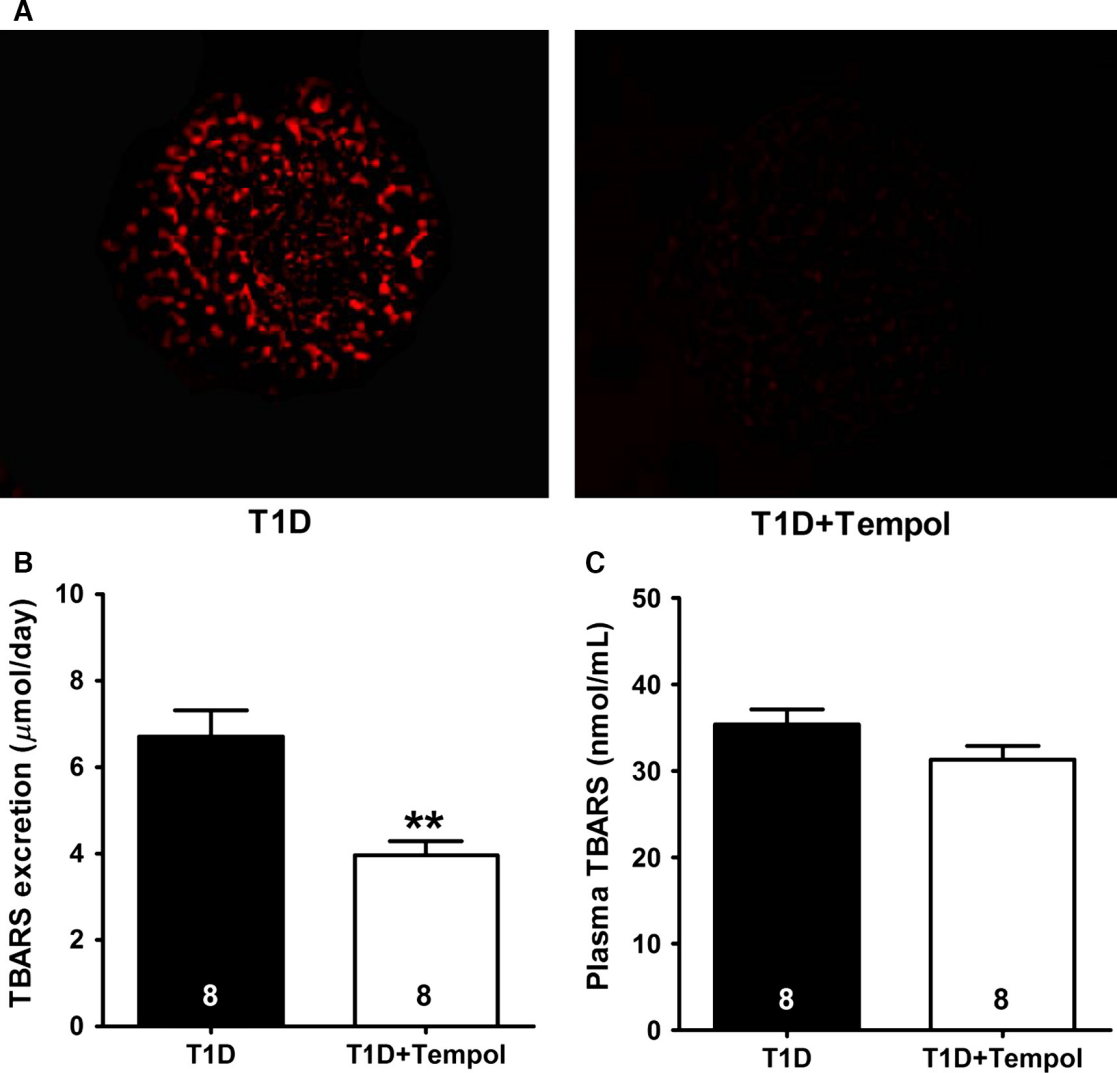
Effect of chronic tempol treatment on oxidative stress indicators in rats with type 1 diabetes mellitus (T1D). (A) Representative images showing dihydroethidium oxidation (red fluorescence), indicative of superoxide production by glomeruli isolated from T1D and T1D+Tempol rats. (B) Urinary excretion of thiobarbituric acid reactive substances (TBARS). (C) Plasma TBARS levels (**P < 0.01 vs. T1D; number of animals shown at bottom of each bar).

Figure 1A shows representative images of DHE staining in glomeruli from T1D and T1D+Tempol rats. Compared with glomeruli isolated from T1D rats, in which DHE fluorescence was prominent, the fluorescence signal was almost completely absent in glomeruli isolated from T1D+Tempol rats. Urinary TBARS excretion was significantly lower in the T1D+Tempol group than in the untreated T1D rats (Fig. 1B), although plasma TBARS did not differ between groups (Fig. 1C). Thus, tempol treatment reduced renal indices of oxidative stress in rats with T1D.

### ET‐1 excretion and renal expression of prepro‐ET‐1

**Figure 2:**
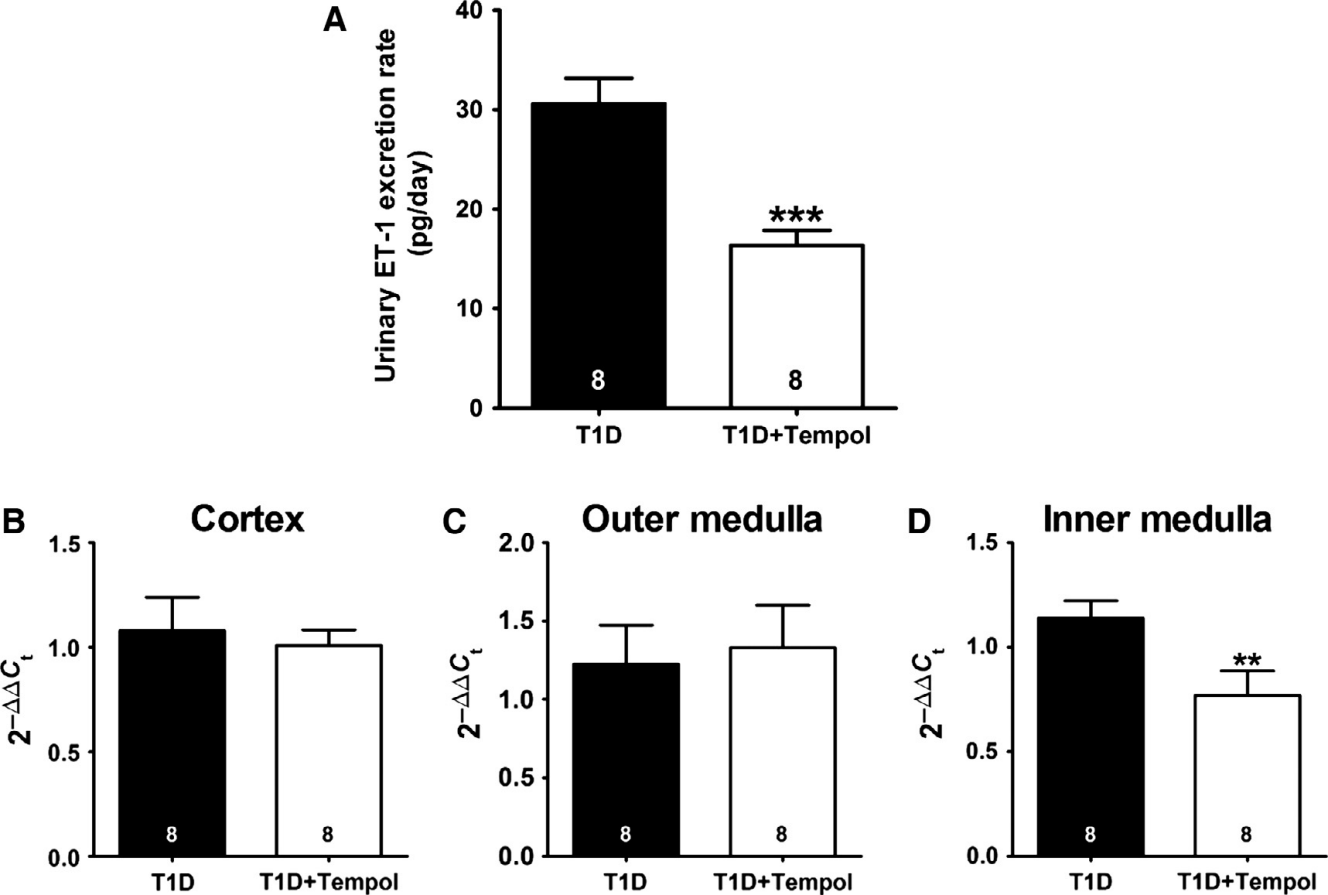
Effect of chronic tempol treatment on the renal endothelin system in rats with type 1 diabetes mellitus (T1D). Shown are urinary endothelin‐1 (ET‐1) excretion (A), and prepro‐ET‐1 mRNA expression measured by RT‐PCR in renal cortex (B), outer medulla (C), and inner medulla (D) for both T1D and T1D+Tempol groups (**P < 0.01 and ***P < 0.001 vs. T1D; number of animals shown at bottom of each bar).

Urinary ET‐1 excretion and inner medullary expression of preproET‐1 mRNA were 40–50% lower in diabetic rats receiving tempol than in untreated T1D rats (Fig. 2). In contrast, expression of preproET‐1 mRNA in renal cortex and outer medulla did not differ between groups.

### Renal protein handling

**Figure 3:**
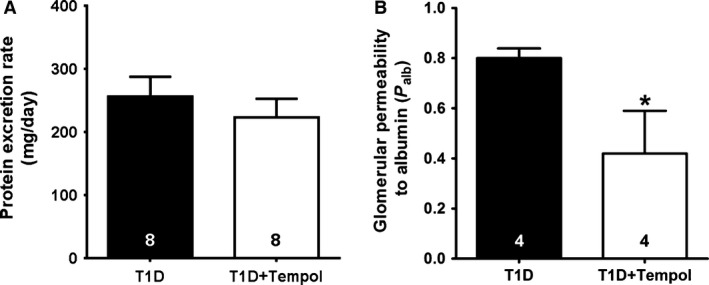
Effects of chronic tempol treatment on urinary protein excretion (A) and glomerular permeability to albumin (B) in diabetic rats. P_alb_ data based on 10–15 glomeruli per animal (*P < 0.05 vs. T1D; number of animals shown at bottom of each bar).

Tempol treatment had no influence on urinary protein excretion (Fig. 3A); however, glomerular P_alb_ in the T1D+Tempol group was approximately 50% lower than that evident in untreated T1D rats (Fig. 3B).

## Discussion

T1D has been shown to increase glomerular P_alb_ (Saleh et al. 2011; Fan et al. 2015) and O_2_^•−^ production (Stanton 2011). Results of this study demonstrate that chronic treatment with the free radical scavenger tempol not only blunts glomerular O_2_^•−^ production and decreases urinary TBARS excretion in rats with T1D, but also reduces glomerular P_alb_ as well as renal ET‐1 synthesis and excretion. Previous studies from our lab revealed that ET‐1 increases the P_alb_ of glomeruli isolated from normoglycemic rats (in vitro) or from chronic ET‐1‐infused rats in vivo (Saleh et al. 2010), and that ET_A_ receptor antagonism prevents the increase in P_alb_ that arises in T1D (Saleh et al. 2011). Our present results extend those observations in support of the hypothesis that oxidative stress in T1D promotes ET‐1 production that, in turn, contributes to glomerular dysfunction.

Several clinical trials indicate that ET‐1 receptor blockade reduces albuminuria in adults with diabetic nephropathy (Gagliardini et al. 2015); however, the mechanism through which the renal ET‐1 system is activated and exerts its pathophysiological effects in T1D is unknown. Urinary ET‐1 excretion, derived from ET‐1 produced by a variety of renal cell types (Abassi et al. 1993; Nakamura et al. 2000), is increased as early as 2 weeks after onset of STZ‐induced T1D in the rat (Sasser et al. 2007), indicating activation of the renal ET system early in the course of the disease. ET_A_ receptor antagonism ameliorates albuminuria and inflammation (but not oxidative stress) for up to 10 weeks in this model (Sasser et al. 2007), indicating that oxidative stress is not the major mechanism through which ET‐1 contributes to the deleterious impact of T1D on the kidney. Rather, our present observation that antioxidant therapy reduces ET‐1 excretion and inner medullary preproET‐1 expression in T1D positions oxidative stress upstream of ET‐1 in provoking renal dysfunction under these conditions, with oxidative stress promoting renal ET‐1 generation. Consistent with this postulate, Chen and co‐workers (Chen et al. 2000) have reported elevated basal production of both superoxide and ET‐1 in glomeruli from rats studied 1 week and 1 month after induction of T1D, and that chronic antioxidant treatment resulted in diminished ET‐1 production.

High glucose concentrations have been reported to enhance endothelial cell ET‐1 secretion (Yamauchi et al. 1990) and to increase ET‐1 gene expression in multiple renal cell types through mechanisms ranging from a direct effect on the ET‐1 promoter to activation of polyADP‐ribose polymerase (PARP), protein kinase C, and/or NF‐*κ*B (Hargrove et al. 2000; Minchenko et al. 2003). O_2_^•−^ and H_2_O_2_ augment, while ROS scavengers reduce, ET‐1 production by mesangial cells (Hughes et al. 1996) and activate the ET‐1 promoter to increase ET‐1 mRNA expression and ET‐1 production by human endothelial cells (Kahler et al. 2000). We speculate that the mechanism of ROS‐dependent ET‐1 production may be related to the consensus sequence for the calcium‐responsive element of the preproET‐1 promoter that is a potential target for oxygen‐derived radicals in inner medullary collecting duct dells, but not endothelial cells (Strait et al. 2007). ROS‐dependent NF‐*κ*B activation may also stimulate the preproET‐1 gene (Geng et al. 1997).

Consistent with Koya et al. (2003), who demonstrated that ROS are generated in vivo by glomeruli 2 weeks after inducing T1D and that this phenomenon can be ameliorated *via* antioxidant treatment, our observations indicate that chronic treatment with the free radical scavenger and superoxide dismutase mimetic, tempol, reduces glomerular O_2_^•−^ levels in animals studied 3 weeks after inducing T1D. Savin's group investigated the role of O_2_^•−^ in mediating glomerular P_alb_ defects induced by various insults (Dileepan et al. 1993); however, they did not examine the in vivo influence of free radicals on P_alb_ nor did they utilize animal models representing chronic kidney diseases such as T1D. Our results indicate that treatment with the free radical scavenger tempol reduces glomerular P_alb_ in rats with T1D. O_2_^•−^ scavenging may impact glomerular P_alb_ by maintaining the function of nitric oxide, which has been shown to preserve low P_alb_ by antagonizing O_2_^•−^ (Sharma et al. 2005). ROS may also alter the properties of podocytes, cytoskeleton, and/or slit‐diaphragm proteins, possibly by lipid peroxidation or production of other mediators (i.e., TNF‐*α*, TGF‐*β*, eicosanoids, other cytokines, or ET‐1), contributing to increased P_alb_. We previously reported evidence of nephrin shedding (increased nephrin excretion and reduced renal nephrin levels) associated with increased P_alb_ 6 weeks after induction of T1D (Saleh et al. 2011). Although nephrinuria was not evident at the 3‐week time point that was utilized in this study (Saleh et al. 2011), we cannot rule out the possibility that other slit diaphragm proteins are involved in evoking the change in P_alb_. Interesting, despite the improvement in P_alb_, tempol had no effect on protein excretion. This observation is consistent with our previous study showing that ET‐1 infusion in nondiabetic rats increases glomerular P_alb_ without influencing urinary excretion of albumin or protein (Saleh et al. 2010), supporting the idea that a rise in P_alb_ precedes actual proteinuria (Mccarthy et al. 2004). Presumably, proximal tubular protein reabsorption is sufficient to temper proteinuria at this early stage of T1D despite alterations in P_alb_.

The tempol treatment regimen employed in this study was exerts an antioxidant effect in numerous animal models, including the spontaneously hypertensive rat (Schnackenberg and Wilcox 1999) and rats with STZ‐induced T1D (Rodriguez et al. 2011; Luan et al. 2012). The renal antioxidant efficacy of tempol in this study was verified by the significant decline in TBARS excretion and the apparent abrogation of renal DHE fluorescence in the T1D+Tempol group compared to the untreated T1D group. Previous in vivo studies employing 1 mmol/L tempol administration via the drinking water have variably reported changes in food and water intake. For example, we and others observed no effect of tempol on food or water intake of normal rats (Luan et al. 2012; Troncoso Brindeiro et al. 2012), while Schackenberg and Wilcox (Schnackenberg and Wilcox 1999) reported increased water intake. Thus, the decline in water intake observed in this study does not likely reflect an adverse impact of tempol on the taste of the drinking water. Similar to this study, Luan et al. (2012) reported that inclusion of 1 mmol/L tempol in the drinking water reduced food and water intake, as well as urine flow, in rats with STZ‐induced T1D. A tempol‐induced decrease in the filtered load of glucose is unlikely to have contributed to the reduced urine flow, water intake, and food intake, as neither blood glucose nor glomerular filtration rate (creatinine clearance) differed between T1D and T1D+Tempol rats. It is possible that tempol reduced the need for increased water intake normally seen with hyperphagia, or that the antioxidant effect of tempol increased proximal tubular solute and water reabsorption (Panico et al. 2009), contributing to the decline in urine flow and the resulting adjustments in water and food intake.

In conclusion, this study provides evidence that in vivo scavenging of free radicals decreases glomerular P_alb_ in early T1D and reduces the renal inner medullary synthesis and urinary excretion of ET‐1. Taken together with the effect of ET_A_ receptor antagonist treatment to prevent the increased P_Alb_ and albuminuria (Sasser et al. 2007; Saleh et al. 2011), while not impacting indices of renal oxidative stress (Sasser et al. 2007), our observations are consistent with the contention that oxidative stress in early T1D promotes activation of the ET‐1 system that, in turn, contributes to glomerular P_Alb_. Further study is necessary to determine whether or not similar events occur in type 2 diabetes.

## Conflict of Interest

None declared.
